# Functional Mobility, Aging, and People With Mental Illness: Issues and Challenges

**DOI:** 10.1093/geroni/igab046.1586

**Published:** 2021-12-17

**Authors:** Michelle Zechner, Ellen Anderson, Kenneth Gill

**Affiliations:** 1 Rutgers, New Jersey, United States; 2 Rutgers, Newark, New Jersey, United States; 3 Rutgers, Piscataway, New Jersey, United States

## Abstract

People with serious mental illness (SMI) are more likely to experience chronic health conditions at younger ages, which increases the risk of premature death. Co-morbid health conditions and risk for premature death are well-studied in the population, however less is understood about the impact of aging and SMI on functional ability. Research suggests that the population walk less and may have lower fitness levels than other populations (Gill et al., 2016). Specific data exploring functional age of people with SMI is sparse. The authors compared published standardized geriatric functional fitness values for people over 65 to baseline values of a community sample of people living with SMI who participated in a community health promotion intervention. The average age of the sample was 50 (SD=11). Three physical functioning measures were used in the comparison to measure physical functioning; the Sit to Stand Test, 6 Minute Walk, and Single Legged Stance. Results indicated significant differences in mean physical functioning values between the sample and standardized geriatric values. The sample performed at levels 20-30 years older than their chronological age. This finding suggests that mental health and aging services may need to adjust interventions, services and methods to improve physical functioning in middle-aged and older adults living with SMI. Premature functional decline impacts community living skills, independent living, housing choice, vocational options, and may impede personal goal attainment. Recommendations for interventions will be offered, as will suggestions for policies targeting services that cross aging and mental health silos.

